# The Utility of Genome-Wide Association Studies in Inherited Arrhythmias and Cardiomyopathies

**DOI:** 10.3390/genes16121448

**Published:** 2025-12-03

**Authors:** Saif Dababneh, Arya Ardehali, Jasleen Badesha, Zachary Laksman

**Affiliations:** 1 Department of Cellular and Physiological Sciences, Faculty of Medicine, University of British Columbia, Vancouver, BC V6T 1Z3, Canada; 2 Cellular and Regenerative Medicine Centre, BC Children’s Hospital Research Institute, Vancouver, BC V5Z 4H4, Canada; 3 Heart Rhythm Services & Center for Cardiovascular Innovation, Division of Cardiology, University of British Columbia, 1033 Davie St, Room 200, Vancouver, BC V6E 1M7, Canada; 4 Faculty of Medicine, University of British Columbia, Vancouver, BC V6T 1Z3, Canada; 5 Centre for Heart Lung Innovation, University of British Columbia, Vancouver, BC V6E 1M7, Canada

**Keywords:** inherited arrhythmias, inherited cardiomyopathies, GWAS, polygenic risk score, hypertrophic cardiomyopathy, dilated cardiomyopathy, long QT syndrome, Brugada syndrome

## Abstract

Inherited arrhythmias and cardiomyopathies are a group of potentially lethal genetic cardiac disorders which are often passed down through generations and pose risks to several family members. While individually rare, these conditions are collectively common and pose significant challenges for clinical management given their variable severity, age of onset, and response to treatments. Earlier genetic analyses revealed crucial insights into the main genetic culprits of these disorders, such as *SCN5A* for Brugada syndrome, and *MYH7* and *MYBPC3* for hypertrophic cardiomyopathy, which have revolutionized diagnosis, risk stratification, and medical management. Nonetheless, issues such as variable expressivity and penetrance, low yield of genetic testing, and relative lack of disease-modifying therapies remain significant hurdles for clinical management. The revolution of genome-wide association studies GWASs has transformed our understanding of inherited arrhythmias and cardiomyopathies, shifting the view of these disorders from a monogenic Mendelian inheritance towards a more complex, often polygenic inheritance with nuanced interplay between genetics and environment. Moreover, GWASs have enabled the quantification of polygenic predisposition to disease using polygenic risk scores, which are often complementary to and independent of monogenic risk. In this review, we highlight how GWASs have transformed the field of inherited arrhythmias and cardiomyopathies, with a particular focus on the polygenic risk scores developed and their clinical utility for the four disorders which have been impacted by GWASs—hypertrophic cardiomyopathy, dilated cardiomyopathy, Brugada syndrome, and long QT syndrome.

## 1. Introduction

Inherited arrhythmias and cardiomyopathies are a diverse group of genetic cardiac disorders which pose significant health risks such as sudden cardiac death and heart failure. Well-known inherited arrhythmias include long QT syndrome (LQTS), Brugada syndrome (BrS), and catecholaminergic polymorphic ventricular tachycardia (CPVT), while well-known cardiomyopathies include hypertrophic cardiomyopathy (HCM), dilated cardiomyopathy (DCM), arrhythmogenic cardiomyopathy (ACM), and restrictive cardiomyopathy (RCM) [1,2]. Though individually rare, these disorders collectively impose a significant health burden due to their potential lethality, the need for lifelong surveillance, and the psychosocial and economic implications for affected families [3,4].

Traditionally, inherited arrhythmias and cardiomyopathies were thought of as solely monogenic disorders following a Mendelian inheritance pattern. Early genetic linkage analysis studies successfully identified many of the major genes involved in the pathogeneses of these disorders, including: *KCNQ1* (OMIM: 607542), *KCNH2* (OMIM: 152427), and *SCN5A* (OMIM: 600163) in LQTS [5,6,7,8]; *SCN5A* in BrS [9], *MYH7* (OMIM: 160760), and *MYBPC3* (OMIM: 600958) in HCM [10,11]; and *PKP2* (OMIM: 602861) in arrhythmogenic right ventricular cardiomyopathy (ARVC)/ACM [10,11,12]. A detailed review of the genetic basis of inherited arrhythmias and cardiomyopathies has previously been published [13,14,15]. These early discoveries not only provided direct diagnostic tests but also transformed our understanding of cardiac physiology and, in some cases, have altered the approach to clinical management [16,17]. For example, the identification of sarcomeric genetic variants in HCM clarified that it is fundamentally a disease of the sarcomere, which recently led to the development of disease-modifying therapies, namely, cardiac myosin inhibitors such as mavacamten, which directly target the sarcomeric dysfunction and hypercontractility seen in HCM [18]. Rapid advancements in genetic testing and next-generation sequencing, from Sanger sequencing and gene panels to whole exome and genome sequencing, further expanded our genetic understanding of inherited arrhythmias and cardiomyopathies, revealing novel genes such as *BAG3* (OMIM: 603883) [19], *FLNC* (OMIM: 102565) [20], and *TMEM43* (OMIM: 612048) [21].

**Figure 1:**
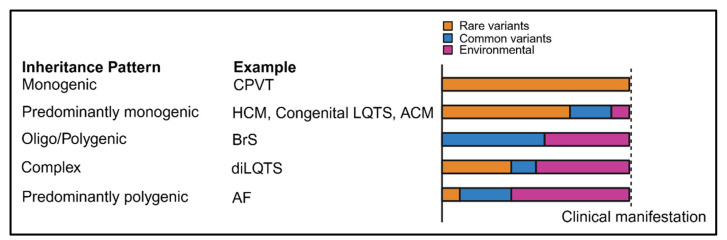
Complex genetic inheritance patterns of arrhythmias and cardiomyopathies. CPVT: catecholaminergic polymorphic ventricular tachycardia, HCM: hypertrophic cardiomyopathy, LQTS: long QT syndrome, ACM: arrhythmogenic cardiomyopathy, BrS: Brugada syndrome, diLQTS: drug-induced long QT syndrome, AF: atrial fibrillation.

Despite the advancements in genomics, the yield of genetic testing in inherited arrhythmias and cardiomyopathies remains limited and varies largely based on the phenotype [16]. For instance, in HCM only 30–40% of patients referred for genetic testing harbor pathogenic variants in sarcomeric genes [22], while only 20–30% of BrS cases can be explained by rare coding variants in *SCN5A* [23]. Moreover, the penetrance and expressivity of disease-causing variants can vary greatly. While *TTN* (OMIM: 188840) truncating variants (*TTN*tvs), which are the most common genetic cause of DCM, are present in 0.5–1% of the population [24,25], only a small minority of this group manifest clinical DCM. In BrS, *SCN5A* loss-of-function variants can manifest as typical BrS type I ECG patterns and malignant arrhythmias in some individuals, whereas other carriers exhibit only subtle conduction delay or remain asymptomatic [26]. These observations underscore the necessity of moving beyond a purely monogenic paradigm for inherited arrhythmias and cardiomyopathies and highlight the existence of genetic mechanisms which are more complex (Figure 1) [27], and which cannot be captured solely using targeted genetic testing for rare coding variants. The various models of inheritance for inherited arrhythmias and cardiomyopathies have been discussed thoroughly elsewhere [27,28].

Genome-wide association studies (GWASs), which involve testing the association of common single nucleotide polymorphisms (SNPs) with phenotypes of interest in large numbers of case and control subjects, has revolutionized genetic discovery through the unbiased identification of loci implicated in disease [29]. While GWASs have traditionally been implemented for common diseases, such as coronary artery disease and atrial fibrillation, given the feasibility and statistical power, the implementation of GWASs for conditions that are relatively more rare, or cardiac parameters that correlate with rare conditions, has provided invaluable insights. In particular, conducting GWASs on cardiac magnetic resonance-derived traits, such as left and right ventricular parameters, and electrocardiogram-derived traits, such as the QT interval, has importantly complemented disease specific GWASs conducted in disease cohorts, such as those for HCM, DCM, and LQTS [28]. These studies have critically expanded our understanding of the complex, genetic architecture of these conditions, providing novel insights into disease pathways that were previously underappreciated or unknown, as well as allowing the development of clinically applicable polygenic risk scores to predict genetic liability regardless of family history or rare pathogenic variant carrier status. In this review, we provide a brief overview of GWASs with a focus on how their application in the field of inherited arrhythmias and cardiomyopathies has advanced our knowledge on a fundamental level and paved the path towards translation of genetic risk prediction into the clinic.

## 2. A Primer on GWAS

The implementation of GWAS has immensely improved our understanding of the genetic basis of inherited cardiac disorders. Unlike traditional linkage and candidate-gene studies, which target rare variants with large effects, GWAS surveys millions of common single nucleotide polymorphisms (SNPs) across the genome to identify loci associated with disease or quantitative traits [30]. By leveraging large cohorts of affected individuals and population controls, GWAS can detect modest effect sizes, revealing how common genetic variation contributes to both susceptibility and phenotypic variability.

The methodological workflow of GWAS has been reviewed extensively elsewhere [30]. Briefly, GWAS involves testing the association of genome-wide SNPs in genotyped case and control participants, often using arrays covering hundreds of thousands of SNPs. GWAS can also be performed on continuous traits (e.g., heart rate, QT interval, and left ventricular ejection fraction) which underlie the phenotypes of rare cardiac disorders. Imputation against reference panels such as the 1000 Genomes Project or Haplotype Reference Consortium extends coverage to millions of additional SNPs, increasing the likelihood of capturing causal loci. Association testing is typically performed under additive models, adjusting for population stratification, age, sex, and other covariates. To minimize false positives, a Bonferroni’s correction is usually applied with a genome-wide significance threshold set at *p* < 5 × 10^−8^. Critical QC steps include filtering for individual and SNP missingness, reported and genetic sex mismatch, Hardy–Weinberg equilibrium deviation and extreme heterozygosity, setting minor allele frequency thresholds, and measuring relatedness and population stratification [31].

A GWAS can provide millions of SNPs, many of which are in the non-coding genome, requiring further narrowing of results to identify the most biologically meaningful signals. Several methods exist to locate important genes, including MAGMA [32], FLAG [33], and transcriptome-wide association studies (TWAS) [34]. Fine-mapping employs statistical tools to narrow the focus down to causal variants from a larger set of variants that are often inherited together (i.e., in linkage disequilibrium), followed by colocalization to link the variant to its closest candidate gene using expression quantitative trait locus data, or linking it to other related traits [35,36].

While early GWASs were limited by small sample sizes and European-only participant samples and lacked power to detect modest effects, the expansion of collaborative consortia, meta-analyses, and biobank datasets has greatly increased discovery potential. This has been particularly transformative for rare inherited cardiac disorders, for which international collaboration is critical for statistical power and representation of diverse populations. While there is no minimum number of cases and controls required to perform a GWAS, a minimum of a 1:1 ratio of cases to controls is recommended, with increasing numbers of controls added minimally beyond a 1:4 ratio [37,38], although this can vary based on minor allele frequencies, disease prevalence, linkage disequilibrium, and the number of SNPs tested. Nonetheless, lack of representation remains a major issue in GWASs. Besides expanding to large and diverse populations, there have been several computational tools utilized to improve transferability of genetic findings across ancestries, such as ancestry-aware fine-mapping (e.g., SuShiE) [39].

Importantly, GWASs in inherited cardiac disorders have revealed several novel risk loci, such as *HEY2* (OMIM: 604674) for BrS, *NOS1AP* (OMIM: 605551) for LQTS, and *FHOD3* (OMIM: 609691) for HCM, revealing critical information about the biological pathways driving these disorders, as discussed in more detail in the following section. [28,40,41] GWASs can also improve drug discovery success by providing genetic evidence for the effect of modulating a gene’s function [42]. Notably, the majority of identified loci lie within noncoding regions, implicating gene regulatory mechanisms, transcription factor binding sites, enhancers, and chromatin loops rather than direct protein-coding alterations. This renders the functional validation of GWAS hits challenging and resource intensive, requiring thorough transcriptomic and epigenomic studies in animal models and, more recently, human stem-cell derived cardiomyocytes [43,44].

## 3. Polygenic Risk Scores—Derivation, Application, and Utility

The development of the polygenic risk score (PRS) represents an extension of GWAS findings, allowing the integration of many common variants into a single quantitative measure of genetic susceptibility. A PRS is calculated by summing the number of risk SNPs carried by an individual, weighted by each SNP’s effect size, as determined from the GWAS. Various statistical methods exist to optimize the PRS, including pruning and thresholding, Bayesian shrinkage approaches, and machine learning-based models, as reviewed more extensively elsewhere [45]. The performance of a PRS depends on several factors, including the size and ancestry of the discovery cohort, the heritability explained by the included SNPs, and the linkage disequilibrium structure of the population. PRSs derived from European-ancestry cohorts often exhibit reduced predictive accuracy when applied to individuals of non-European ancestry, highlighting a critical limitation for clinical implementation [46].

PRSs are continually updated as both the data and the methods involved continue to evolve. As GWAS sample sizes increase, effect size estimates become more precise, and new loci are discovered, which alters the set of variants included in a score. Improvements in imputation, cross-ancestry analyses, and fine-mapping also refine the determination of which variants are most biologically relevant [47]. With the relatively limited PRSs generated based on mixed ancestry data, there have been several tools developed to develop more ancestrally appropriate PRSs from European GWAS datasets, including PRS-CSx and CT-SLEB [47,48], although these also remain limited and cannot be automatically applied to all ancestries across all diseases [49].

Determining when a PRS is ready for clinical use requires evidence beyond statistical significance. Gold-standard metrics include discrimination (e.g., area under the curve, odds ratios per standard deviation), calibration, improvement in predictive value over standard models, and validation in external datasets [50,51]. Key reporting parameters include odds ratio/hazard ratio per one-standard-deviation change in PRS, coefficient of determination (R^2^), C-index, area under the receiver operator curve, and net reclassification improvement [52,53]. Crucially, PRSs must be validated across diverse ancestries to ensure equitable performance. The ultimate test would be prospective clinical trials showing that PRS-guided management improves patient outcomes. Until then, PRSs for inherited arrhythmias and cardiomyopathies remain promising research tools with emerging clinical potential.

## 4. Applications of GWAS in Inherited Arrhythmias and Cardiomyopathies

**Figure 2:**
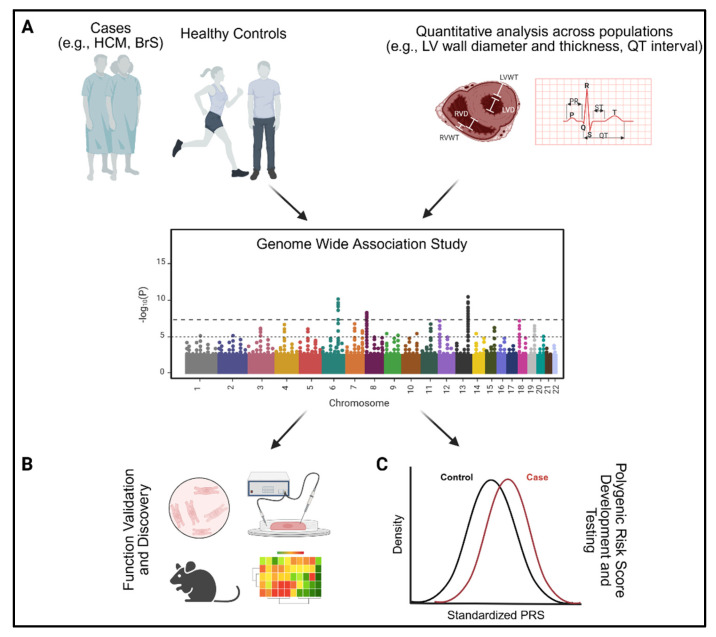
Applications of GWAS in inherited arrhythmias and cardiomyopathies. (**A**) Case–control studies and large-scale analysis of quantitative traits relevant to inherited arrhythmias and cardiomyopathies both contribute to genome-wide association studies (GWAS) applicable to patients with inherited cardiac conditions. (**B**) Following identification of relevant loci, GWAS data can be leveraged to uncover novel disease pathways and validate targets using preclinical models and functional assays. Preclinical models include animal models (e.g., transgenic mouse models, zebrafish) while emerging in vitro models include human induced pluripotent stem cell-derived cardiomyocytes and organoids. These models enable the examination of the molecular and functional consequences of genetic variants in a cardiac context, as well as the impacts of variants on gene regulation using methods such as luciferase, chromatin accessibility, and transcription factor binding assays. (**C**) GWAS data also contributes to the development of polygenic risk scores for clinical risk prediction and stratification in independent datasets. HCM: hypertrophic cardiomyopathy, BrS: Brugada syndrome. LV: left ventricle, LVWT: left ventricular wall thickness, LVD: left ventricular diameter, RV: right ventricle, RVWT: right ventricular wall thickness, RVD: right ventricular diameter, GWAS: genome-wide association study, PRS: polygenic risk score.

Given the rarity of inherited arrhythmias and cardiomyopathies, there are relatively fewer GWASs conducted in inherited cardiac disease cohorts, compared to more common pathologies such as coronary artery disease or atrial fibrillation. However, the few GWAS efforts that have been implemented for HCM, DCM, BrS, and LQTS and related cardiac traits (e.g., LV wall thickness, QT interval) have provided critical insights into novel disease pathways and are rapidly enabling the generation of disease-specific polygenic risk scores (Figure 2). In this section, we highlight how GWASs for each of these disorders have advanced our understanding of the pathophysiologies of these disorders and the ongoing polygenic risk score efforts in disease prediction and risk stratification. A summary of the polygenic risk score studies relevant to these disorders, outlining the predictive ability of each PRS, number of SNPs in each PRS, ancestry used in the training and validation sets, and comparison of the genetic score performance with clinical models when available, can be found in Table 1 for inherited cardiomyopathies and Table 2 for inherited arrhythmias. GWASs on more rare arrhythmias and cardiomyopathies, such as CPVT and arrhythmogenic right ventricular cardiomyopathy (ARVC), remain lacking given the challenges in conducting sufficient case–control studies and may provide limited utility in strongly monogenic diseases, such as CPVT. Addressing this gap may be possible in the near future as international collaborations continue to accelerate.

### 4.1. HCM

Hypertrophic cardiomyopathy (HCM) is the one of the most common inherited cardiomyopathies, with an estimated prevalence of ~1 in 500. HCM is characterized by left ventricular hypertrophy—which can be often obstructive—myocyte disarray, and a predisposition to ventricular arrhythmias and sudden cardiac death. HCM has traditionally been viewed as a monogenic disorder caused by rare, high-penetrance variants in sarcomeric genes, the three most common being *MYH7*, *MYBPC3*, and *TNNT2* (OMIM: 191045) [54]. However, the yield of genetic testing remains between 30–40%, and carriers of the same rare pathogenic HCM variant can demonstrate considerable phenotypic heterogeneity and variable outcomes [55].

#### 4.1.1. Novel Insights from GWAS

The implementation of GWAS for HCM has been instrumental in addressing important knowledge gaps through identifying common variants that contribute to HCM susceptibility and modulate left ventricular wall thickness. HCM GWAS highlights common variations in traditional HCM risk genes such as *MYH7* but also identifies novel sarcomeric and non-sarcomeric loci and disease pathways contributing to HCM pathophysiology [55,56,57]. For instance, a large GWAS meta-analysis integrating exome sequencing and array-based genotyping in 5900 HCM cases validated the associations near *FHOD3*, a gene involved in actin filament formation and sarcomere assembly [57]. Additional loci implicate transcription factor regulators (e.g., *ALPK3*; OMIM: 617608), calcium handling proteins (e.g., *PLN*; OMIM: 172405), and structural proteins (e.g., *ACTN2*; OMIM: 102573, *CSRP3*; OMIM: 600824), highlighting the interplay between sarcomeric and non-sarcomeric pathways in shaping HCM risk.

#### 4.1.2. Polygenic Risk Scores

Based on imaging data, Ning et al. generated a PRS for 12 left ventricular regional wall thickness (LVRWT) traits using a GWAS conducted in 42,194 European-ancestry UK Biobank participants with cardiac magnetic resonance (CMR) imaging data [58], with the most predictive score for HCM based on inferoseptal thickness at end systole (ES-IS). PRS_LVRWT_ significantly predicted incident HCM (HR per one SD of 1.69) while showing no meaningful associations with other cardiovascular diseases, such as hypertension, myocardial infarction, or stroke. Although the PRS remained an independent predictor when adjusting for clinical factors, its standalone predictive power was modest, with only slight improvement achieved when added to clinical models.

One of the first HCM-specific PRSs was developed by Harper et al., and included 27 independent HCM-associated SNPs based on a mixed-ancestry GWAS meta-analysis of two hospital/clinic-based cohorts (HCMR and BRRD) [59]. Validation was performed in three independent cohorts from Genomics England, the Royal Brompton Hospital, and the Netherlands. PRS_HCM_ significantly predicted the odds of HCM and was also associated with greater LV wall thickness in sarcomere variant carriers. Notably, PRS_HCM_ performed similarly across ancestry groups and showed an OR of 1.73 per SD increase in validation cohorts, and had predictive value independent of rare HCM variant status. Using this same GWAS, another group evaluated the utility of HCM_PRS_ in two large biobanks (UKB and MGB), demonstrating its ability to predict HCM (OR 1.18 per SD) and greater LV wall thickness in the general population, independent of other clinical factors such as age, sex, blood pressure, and body mass index [60]. However, it did not predict other cardiac outcomes like DCM, arrhythmia, or heart failure in participants without HCM. When compared to baseline clinical models, the addition of the PRS modestly but significantly improved prediction.

Most recently, Zheng et al. developed polygenic risk scores for hypertrophic cardiomyopathy using a European-ancestry GWAS meta-analysis of 5900 cases and 68,359 controls from seven cohorts, combined with multi-trait analysis incorporating CMR traits such as LV concentricity, end-systolic volume, and circumferential strain [61]. The PRS was tested in both population-based (UK Biobank) and clinically ascertained cohorts (100,000 Genomes Project, Erasmus MC, and Royal Brompton Hospital). It predicted HCM risk in the general population, penetrance in rare-variant carriers, and adverse cardiovascular outcomes (including death) in diagnosed patients, and correlated with imaging features typical of HCM, while showing no association with atrial fibrillation and an inverse relationship with heart failure. The PRS was an independent predictor after accounting for rare-variant status, with an OR per SD of 2.34 in the general population, 2.35 in sarcomere-positive carriers, and 2.15 in sarcomere-negative carriers. Cross-ancestry analyses showed reduced performance outside European populations, consistent with the GWAS training dataset.

### 4.2. DCM

Dilated cardiomyopathy (DCM) is characterized by ventricular dilation and systolic dysfunction, often leading to heart failure, ventricular arrhythmias, and sudden cardiac death. DCM has an estimated prevalence of between ~1 in 250 and ~1 in 500 [62]. DCM exhibits substantial genetic and etiological heterogeneity, with contributions from rare variants in structural genes such as *TTN*, *LMNA* (OMIM: 150330), and *RBM20* (OMIM: 613171); common polygenic variation; and environmental triggers such as inflammation and infection. *TTN* truncating variants (*TTN*tvs) are the most common genetic form of DCM and are highly prevalent in DCM patients, but are also present in apparently healthy people in the general population, with only a subset of *TTN*tv carriers developing disease, highlighting the importance of additional modifiers [23,24].

#### 4.2.1. Novel Insights from GWAS

Several GWASs conducted either in DCM cohorts or DCM-relevant CMR traits (e.g., LVEF) have elucidated several loci that influence both susceptibility and progression in DCM [58,63,64,65]. Some novel loci include *BAG3*, which encodes a co-chaperone involved in protein quality control and sarcomere maintenance. Rare pathogenic variants in *BAG3* cause a high penetrance and progressive DCM phenotype [66], while common variants, such as p.C151R, act as genetic modifiers to influence DCM penetrance and expressivity, even in *TTN*tv carriers [67]. Another notable gene is *HSPB7* (OMIM: 610692), a heat shock protein required for the intercalated disc integrity critical for heart development [68]. Notably, some HCM GWAS-associated loci are also associated with DCM, including *MYBPC3*, *ALPK3* (OMIM: 617608), and *FHOD3*, emphasizing that the same disease pathways affect HCM and DCM inversely. In particular, Tadros et al. demonstrated how SNPs in the same genes show inverse associations between HCM and DCM, whereby SNPs that increase risk for DCM decrease risk for HCM, and vice versa [56]. This has important therapeutic implications, particularly given the demonstrated efficacy of cardiac myosin inhibitors (e.g., mavacamten) for HCM, which suggests that targeting the inverse pathway via cardiac myosin activators (e.g., omecamtiv mecarbil) may be a promising disease-modifying therapy for DCM [69].

#### 4.2.2. Polygenic Risk Scores

Imaging-based studies have significantly shaped our understanding of the polygenic contributions to the DCM phenotype, like in HCM. The earliest study by Aung et al. developed PRSs for six cardiac MRI-derived LV traits, namely, LV end-diastolic volume (LVEDV), LV end-systolic volume (LVESV), LV stroke volume (LVSV), LV ejection fraction (LVEF), LV mass (LVM), and LV mass-to-volume ratio (LVMVR), using European-ancestry UK Biobank participant imaging data [70]. Heart failure was predicted by several PRSs, namely PRS_LVEDV_, PRS_LVESV_, PRS_LVEF_, and PRS_LVMR_, even after adjustment for demographic and clinical risk factors. Increasing PRS_LVEDV_ and PRS_LVESV_ values were associated with increased heart failure risk, while increasing PRS_LVEF_ values were protective. Interestingly, PRS_LVM_ was not predictive, and PRS_LVSV_ had limited utility due to lack of significant loci. Shortly after, Pirruccello et al. generated a PRS for LV end-systolic volume indexed to body surface area (LVESVi), a CMR-derived measure related to heart structure and function [71]. PRS_LVESVi_ consisted of 28 SNPs identified from a GWAS in 36,041 UK Biobank participants without cardiovascular disease at baseline. Among several PRSs developed for different cardiac traits, the PRS_LVESVi_ showed the strongest and most significant association with DCM in both cross-sectional and prospective analyses, with a HR of 1.58 per SD increase. PRS_LVESVi_ predicted DCM independently of clinical factors such as age, sex, and ancestry, but the study did not directly compare its predictive performance to established clinical models. Overall, PRS_LVESVi_ provides a genetic risk measure that complements clinical data for better stratification of DCM risk.

Right ventricular (RV) imaging data have also informed PRS development for DCM. Pirruccello et al. conducted an RV trait GWAS in a predominantly European population-based cohort (UK Biobank), yielding approximately 1.1 million SNPs, which were used to generate RV trait PRSs [72]. Notably, the most significant PRS was for right ventricular ejection fraction (RVEF), which strongly and independently predicted incident DCM (HR 1.33 per one SD decrease) and remained significant after adjusting for a left ventricular PRS (HR 1.21 per SD decrease). The findings were replicated in external biobanks, including the hospital-based MGB Biobank and BioBank Japan.

The first DCM-specific PRS (PRS_DCM_) was developed by Garnier et al., and included four SNPs from the DCM associated loci *BAG3*, *HSPB7*, *SLC6A6* (OMIM: 186854), and *SMARCB1* (OMIM: 601607) [73]. PRS_DCM_ showed a clear association with DCM risk, whereby individuals with eight risk alleles had an OR of 3.34, while those with one risk allele had a ~5-fold decreased risk (OR 0.21) compared to the median group. Secondary analyses showed that PRS_DCM_ was significantly associated (OR 1.53) with left ventricular end-diastolic diameter (LVEDD) but was only borderline associated with prognosis (cardiac death or transplant). This PRS_DCM_ was generated and tested entirely in European-ancestry cohorts drawn from several European countries and the US, with no mixed-ancestry data included. Overall, as the first DCM trait-specific PRS, this study demonstrated that polygenic risk meaningfully contributes to DCM, but this finding requires further validation and integration with clinical factors to determine clinical utility.

Most recently, the largest DCM trait-specific PRS was developed using GWAS and multi-trait analysis (MTAG) summary statistics [65]. PRS_DCM_ was tested across several hospital/clinic-based cohorts (e.g., Amsterdam UMC, HERMES, and MVP) and population biobanks (e.g., UK Biobank, FinnGen, All of Us, and MGB). PRS_DCM_ significantly predicted DCM across European, African, and Admixed-American ancestries and was an independent predictor of systolic heart failure in multiple contexts (including after atrial fibrillation, hypertension, and myocardial infarction), remaining predictive even after excluding DCM/NICM cases. MTAG-derived PRS generally outperformed GWAS-only PRS across ancestries in DCM prediction, with OR per SD increases of 1.73, 1.61, and 1.34 in individuals of European ancestry, African ancestry, and Admixed-American ancestry, respectively. PRS_DCM_ remained predictive regardless of rare pathogenic variant status, although more predictive in genotype-negative cases (OR 2.14 per SD increase vs. 1.48 in genotype-negative and genotype-positive, respectively). Notably, PRS_DCM_ improved discrimination and calibration metrics when compared to baseline clinical models. Table 1 summarizes the main PRS studies in HCM and DCM.

**Table 1:** Summary of polygenic risk score studies for inherited cardiomyopathies and related traits.

Study (y)	Discovery (n)	Validation/Test Dataset (n)	Participant Ancestry	PRS	SNPs in PRS	PRS HR/OR (95% Cl), Including Variables Adjusted for	AUC/C Statistic (95% Cl): Clinical Risk Tool or PRS Alone vs. PRS + Clinical Risk Tool	Main Findings
Ning et al. (2023) [59]	42,194	439,981	European	12 LVRWT parameters	6345	HR_ES-IS_ 1.69 (95% CI = 1.33–2.15), no variables adjusted for reported.	C statistic 0.67 for clinical risk model (no 95% CIs reported)	PRSs derived from LVRWT traits are associated with incident HCM.
							C statistic range of 0.67–0.69 for PRS + clinical risk model (no 95% CIs reported)	
Harper et al. (2021) [60]	50,266	GeL—35,935 RBH—1570 Netherlands—3092	Mixed-ancestry	HCM	27	OR per SD 1.73 (95% CI 1.63–1.83), adjusted for first ten principal components, age and gender.	N/A	The PRS predicts the odds of HCM and was associated with greater LV wall thickness in sarcomere variant carriers in a validation meta-analysis of three independent HCM cohorts.
Biddinger et al. (2022) [61]	Used Harper et al. (2021) study	UKB—184,511 MGB—30,716	Largely European (Mixed-ancestry)	HCM	27	HR per SD 1.18 (95% CI, 1.12–1.24), adjusted for age, sex, genotyping array, and PCs 1–5.	AUC 0.670 (95% CI 0.635–0.705) for age, sex, and PCs 1–5	The PRS was associated with increased odds of HCM in the general population.
							AUC 0.706 (95% CI 0.669–0.742) for PRS + co-variates	
Zheng et al. (2025) [62]	HCM GWAS—74,259 Multi-trait GWAS—36,083 Multi-trait	UKB—376,730 (EUR) + 16,349 (non-EUR) GeL—683 EMC—214 RBH—440	Largely European (Mixed-ancestry)	HCM + Multitrait (LVconc, LVESV, strain_circ_)	PGS_GWAS_ = 376,730 SNP predictors PGS_MTAG_ = 374,113 SNP predictors	GWAS: 1.97 OR per SD (95% CI 1.81–2.15) MTAG: OR per SD 2.34 (95% CI 2.12–2.59), adjusted for age, age^2^, sex and first ten genetic PCs.	N/A	The PRS predicted HCM risk in the general population, penetrance in rare variant carriers, and adverse cardiovascular outcomes (including death) in diagnosed patients, and correlated with imaging features typical of HCM.
Aung et al. (2019) [71]	16,923	4383	European	LVEDV LVESV LVEF LVMVR LVSV LVM	Not specified	LVEDV → HR 1.18 (95% CI 1.03–1.36) LVESV → HR 1.32 (95% CI 1.15–1.51) LVEF → HR 0.79 (95% CI 0.69–0.90) LVMVR → HR 0.76 (0.66–0.88) Models adjusted for age, sex, BMI, SBP and DBP corrected for anti-hypertensive medication use, smoking status, regular alcohol use, dyslipidemia, diabetes, and first 15 genetic PCs.	N/A	PRSs for LVEDV, LVESV, LVEF, and LVMVR significantly predicted heart failure after adjustment for age, sex, body size, and standard cardiovascular risk factors, confirming they were independent predictors.
Pirruccello et al. (2020) [72]	36,041	MESA—2184 BBJ—19,000	Discovery: European Validation: Multi-ancestry	LVEDV LVEDVi LVESV LVESVi LVEF SV SVi	28 (LVESVi)	HR per SD 1.58 (95% CI 1.43–1.76), adjusted for sex, genotyping array, the first five PCs, and the cubic basis spline of age at enrollment.	N/A	The PRS for LVESVi significantly predicted future DCM events, indicating that higher genetically mediated LVESVi was linked to a higher DCM risk.
Pirruccello et al. (2022) [73]	41,135	UKB—359,899 MGB—23,386 BBJ—154,450	European	RV Parampara RA measurements PA measurements	1,117,425	HR per SD 1.33 (95% CI not reported), adjusted for covariates including sex, the cubic basis spline of age at enrollment, the interaction between the cubic basis spline of age at enrollment and sex, the genotyping array, the first five PCs of ancestry and the cubic basis splines of height (cm), weight (kg), body mass index (BMI) (kg·m^–2^), diastolic blood pressure, and systolic blood pressure.	N/A	The most significant PRS was for right ventricular ejection fraction (RVEF), which strongly and independently predicted incident DCM.
Garnier et al. (2021) [74]	7159	1547	European	DCM	4	OR per SD 3.34 (95% CI 1.87–6.00) for patients with 8 risk alleles OR per SD 0.21 (95% CI 0.06–0.77) for patients with 1 risk allele.	N/A	The PRS showed a clear association with DCM risk: individuals with eight risk alleles had about a 3-fold increased risk, while those with one risk allele had a 5-fold decreased risk compared to the median group.
Jurgens et al. (2024) [66]	955,733	1,448,963	European, African, and Admixed-American ancestries	DCM	DCM: 1,068,761 –1,098,677 MTAG-DCM: 1,038,394–1,075,760	DCM trait PRS: OR per SD 1.93 (95% CI 1.79–2.07) MTAG PRS: OR per SD 1.73 (95% CI 1.61–1.86)	GWAS DCM AUC 0.64 (95% CI 0.62–0.66) for PRS w/o covariates MTAG-DCM AUC 0.67 (95% CI 0.65–0.68) for PRS w/o covariates	The PRSs significantly predicted DCM across European, African, and Admixed-American ancestries and were independent predictors of systolic heart failure (HF) in multiple contexts.
							GWAS DCM AUC 0.71 (95% CI 0.69–0.73) for PRS w/covariates MTAG-DCM AUC 0.73 (95% CI 0.71–0.75) for PRS w/covariates	

### 4.3. BrS

Brugada syndrome (BrS) is a primary cardiac channelopathy characterized by ST-segment elevation in the right precordial leads and a predisposition to lethal ventricular arrhythmias. Classically, BrS is caused by rare loss-of-function variants in *SCN5A*. However, *SCN5A* variants only explain ~30% of cases, suggesting a large role for additional genetic contributions and environmental factors [74,75].

#### 4.3.1. Novel Insights from GWAS

BrS provides a clear example of GWASs elucidating disease mechanisms beyond rare pathogenic variants. While *SCN5A* variants explain only up to 30% of cases, the largest GWAS, of over 2800 BrS patients, has identified twenty-one independent genome-wide significant SNPs across twelve loci, ten of which were previously unreported [76]. The most common hot spot was within the *SCN5A*–*SCN10A* locus, consistent with the predominant role of sodium channels in disease pathogenesis. However, additional loci were located near cardiac transcription factor genes (e.g., *HEY2*) and genes involved in microtubule and myofilament function (e.g., *MAPRE2*; OMIM: 605789), which have recently been supported by functional validation in preclinical models as playing a role in regulating sodium current density and dynamics [77,78]. Through GWASs, BrS is now thought of as an oligogenic disease, with polygenic risk-based inheritance being a common form.

#### 4.3.2. Polygenic Risk Scores

The first study to test the utility of PRSs for BrS was conducted by Tadros et al., whereby three relevant PRSs, those for PR interval (PRS_PR_), QRS duration (PRS_QRS_), and BrS diagnosis (PRS_BrS_), were generated using prior GWASs with 44, 26, and 3 SNPs, respectively [79]. Here, the authors sought to test whether any of the three PRSs would predict cardiac electrical responses to sodium-channel blockade during ajmaline testing, which is a pharmacological test used to diagnose BrS, in 1400 consecutive patients at Amsterdam UMC. While PRS_PR_ was associated with PR slope in univariable analysis, it was not an independent predictor after adjusting for baseline PR and sex; PRS_QRS_ and age independently predicted QRS slope; and PRS_BrS_, alongside baseline QRS, Type II/III BrS pattern, and family history of BrS, independently predicted ajmaline-induced Type I BrS ECG. PRS_BrS_ demonstrated an OR of 1.141 per one SD increase in the full cohort and 1.159 when excluding *SCN5A* variant carriers. PRS_BrS_ alone yielded a C-statistic of 0.68, which improved to 0.741 when combined with clinical variables, with thresholds achieving up to 95% specificity or 99% sensitivity. Building upon the same three SNP-based PRS_BrS_, Wijeyeratne et al. tested the clinical utility of this PRS in *SCN5A* genotype-positive and genotype-negative BrS patients, recruited from 16 hospital or clinic-based centers worldwide [22]. Remarkably, PRS_BrS_ predicted the presence of spontaneous or drug-induced type 1 ECG pattern (i.e., BrS phenotype) in both *SCN5A* variant carriers and genotype-negative relatives, with ORs per additional risk allele of 1.46 in the total cohort, 1.25 in genotype-positive individuals, and 2.71 in genotype-negative relatives. Effect sizes varied by variant type, being highest for loss-of-function *SCN5A* variants (OR = 5.18), moderate for E1784K-*SCN5A* (OR = 1.49), and nonsignificant for other missense variants (OR = 0.88). Individuals with ≥4 risk alleles had markedly increased odds of BrS, especially in genotype-negative relatives (OR = 22.29).

A larger BrS GWAS was later conducted by Barc et al. in 2820 BrS cases and 10,001 controls; this enabled the development of a 21 independent SNP PRS_BrS_ [76]. This PRS was shown to better predict BrS diagnosis in *SCN5A* genotype-negative individuals compared to genotype-positive individuals, and better predict spontaneous Type I BrS ECG compared to drug-induced Type I BrS ECG. Notably, PRS_BrS_ did not predict lethal arrhythmic events in BrS cases, but was positively associated with related phenotypes such as atrioventricular conduction disorders (OR 1.16 per SD increase) and increasing QRS duration and negatively associated with QT duration.

More recently Ishikawa et al. developed an improved PRS_BrS_ using 17 independent SNPs from a cross-ancestry meta-analysis of European and Japanese GWASs [80]. Here, PRS_BrS_ strongly and independently predicted BrS diagnosis (OR 2.12 per SD) but did not predict lethal arrhythmic events. Although PRS_BrS_ was not formally compared to full clinical models, it showed marked case–control discrimination, with the top 2.5% having over 6-fold greater odds of BrS versus the middle quintile. Interestingly, PRS_BrS_ based on European-ancestry data alone (21 SNPs) was still a robust predictor in Japanese patients, suggesting that BrS polygenic underpinnings are largely shared across ancestries.

### 4.4. LQTS

Long QT syndrome (LQTS) is the most common inherited channelopathy, characterized by a prolonged QT interval on the ECG due to abnormally elongated and heterogenous ventricular repolarization, which predisposes to lethal arrhythmias such as Torsades de Pointes. LQTS types are classified based on genotype, with the main three being LQT1 (*KCNQ1*), LQT2 (*KCNH2*), and LQT3 (*SCN5A*), with genotype greatly influencing medical management and risk stratification approaches [81]. Despite the identification of high-penetrance variants in a large portion of LQTS patients, clinical expression is highly variable, even within families carrying the same variant.

#### 4.4.1. Novel Insights from GWAS

GWASs investigating QT interval duration have identified several risk loci influencing ventricular repolarization, implicating ion channels such as *KCNE1* and *KCNQ1*, but also other targets including transcription factors (e.g., *TBX5*; OMIM: 601620) and metabolic regulators (e.g., *NOS1AP*) [82,83]. A GWAS of 1656 unrelated LQTS cases of European and Japanese ancestry and 9890 controls revealed robust correlation between LQTS genetic susceptibility and the genetics of QT interval in the general population, suggesting robust genetic overlap between the genetics of the QT interval in the general population and LQTS as a disease entity [82]. Notably, variations in the *NOS1AP* locus, which encodes nitric oxide synthase 1 adaptor protein, have emerged as prominent genetic modifiers of QTc (i.e., heart rate-corrected QT interval) prolongation and arrhythmic risk in the general population and in LQTS rare pathogenic variant carriers. The discovery of the *NOS1AP* as a prominent risk locus has resulted in several functional studies confirming the biological role of *NOS1AP* in cardiac repolarization, through regulation of NOS1 (OMIM: 163731) activity, using human stem-cell derived cardiomyocytes and mouse models [84,85]. Similar loci have also been implicated in GWASs on drug-induced LQT, which is a disease entity characterized by marked QT prolongation in response to potentially QT-prolonging medications, particularly in those genetically predisposed to LQT [86].

#### 4.4.2. Polygenic Risk Scores

The first PRS relevant for LQTS was developed by Strauss et al., and included 61 QT interval-associated SNPs. This study demonstrated that PRS_QT_ was robustly associated with drug-induced QTc prolongation, explaining up to 30% of diLQT, and also predicted drug-induced Torsades de Pointes [86]. Turkowski et al. examined the utility of the Strauss et al. 61-SNP PRS in a large, hospital-based cohort at the Mayo Clinic that included both probands and genotype-positive family members with LQT1, LQT2, or LQT3 [87]. PRS_QT_ predicted higher QTc values in probands and those with prolonged QTc but did not predict symptomatic status or serious arrhythmic events. In multivariate analysis, PRS_QT_ remained an independent predictor of proband status but not symptoms, with resting QTc emerging as the strongest predictor of both. Lahrouchi et al. tested the utility of a 68-SNP based PRS_QT_ in LQTS patients and found PRS_QT_ to be higher in LQTS cases relative to controls, particularly in genotype-negative LQTS cases, suggesting a stronger polygenic basis in genotype-negative LQTS [82].

A much larger study was later conducted by Nauffal et al., developing a PRS_QTc_ derived from a QTc GWAS performed in 84,630 UK Biobank participants, which yielded 1,110,494 SNPs [88]. PRS_QTc_ utility was tested in the multi-ancestry TOPMed cohort, showing a significant, independent association with QTc duration: each decile increase in PRS corresponded to a 1.4 ms QTc prolongation, and individuals in the top decile had an average 8.7 ms longer QTc, similar to the effect of monogenic rare variants. While PRS_QTc_ stratified QTc risk across ancestries and improved prediction beyond traditional clinical factors, predictive power was lower in African-ancestry participants. Notably, however, approximately 75% of individuals with marked QTc prolongation lacked either high polygenic risk or rare pathogenic variants, highlighting the roles of environmental and non-genetic risk factors.

Validating the utility of PRSs in diLQT has also been a focus of several studies. Lancaster et al. developed a 465,399 SNP-polygenic risk score (PRS) for baseline QTc (PRS_QTc-baseline_), reflecting the QT interval corrected for heart rate before any drug exposure, and aimed to test its ability to predict QTc prolongation following a standardized sotalol challenge in 978 healthy, European-ancestry volunteers from the GENEREPOL study [89]. The main outcomes were the change in QTc at three hours (ΔQTc) and incidence of extreme QT prolongation (ΔQTc ≥ 60 ms). PRS_QTc-baseline_ predicted both the magnitude of ΔQTc and the likelihood of extreme response but not post-sotalol QTc prolongation (QTc > 500 ms). Importantly, PRS_QTc-baseline_ remained an independent predictor after adjustments for age, sex, plasma sotalol concentration, plasma potassium, and genetic ancestry. Another two studies have similarly reported the significant utility of PRS_QT_ in predicting drug-induced LQT across various cohorts and ancestries, although the score remains less robust for non-Europeans given the lack of multi-ancestry data [90,91]. Table 2 summarizes the main PRS studies in BrS and LQTS.

**Table 2:** Summary of polygenic risk score studies for inherited arrhythmias and related traits.

Authors (y)	Discovery (n)	Validation/Test Dataset (n)	Participant Ancestry	PRS	SNPs in PRS	PRS HR/OR (95% Cl), Including Variables Adjusted for	AUC/C Statistic (95% Cl): Clinical Risk Tool or PRS Alone vs. PRS + Clinical Risk Tool	Main Findings
Tadros et al. (2019) [80]	1368	N/A	European	PR interval (PRS_PR_) QRS duration (PRS_QRS_) Brugada syndrome (PRS_BrS_)	PRS_PR_: 44 PRS_QRS_: 26 PRS_BrS_: 3	PRS_BrS_ OR per SD 1.14 (95% CI 1.10–1.18), full ancestry cohort PRS_BrS_ OR per SD 1.16 (95% CI 1.12–1.20), when excluding *SCN5A* variant carriers	C statistic 0.68 for PRS_BrS_ (95% CI 0.65–0.71)	PRS_BrS_, alongside baseline QRS, Type II/III BrS pattern, and family history of BrS, independently predicted ajmaline-induced Type I BrS ECG.
							C statistic 0.74 for PRS_BrS_ + Family history for BrS and Baseline ECG data (95% CI 0.71–0.77)	
Wijeyeratne et al. (2020) [23]	Tadros et al. (2019) study	312	Mixed ancestry	BrS	3 SNPs, 6 high-risk alleles	OR per risk allele 1.46 (95% CI 1.11–1.94) OR for BrS ≥ 4 risk alleles 4.15 (95% CI 1.45–11.85) for BrS phenotype	N/A	The PRS predicted the presence of spontaneous or drug-induced type 1 ECG pattern (i.e., BrS phenotype) in both *SCN5A* mutation carriers and genotype-negative relatives.
Barc et al. (2022) [77]	12,821	N/A	European	BrS	21	OR per SD 1.16 (95% CI 1.10–1.21) for atrioventricular conduction disorders	N/A	The PRS better predicted BrS diagnosis in *SCN5A* genotype-negative individuals compared to genotype-positive individuals, and better predicted spontaneous Type I BrS ECG compared to drug-induced Type I BrS ECG.
Ishikawa et al. (2024) [81]	Japanese cohort = 2574 Meta analysis Japanese and European GWAS = 15,395	N/A	Japanese and European	BrS	17	OR per SD 2.12 (95% CI 1.94–2.31) risk of BrS in the Japanese cohort	N/A	The PRS strongly and independently predicted BrS presence, but did not predict lethal arrhythmic events.
Strauss et al. (2017) [87]	22	987	Mixed-Ancestry	QT	61	N/A	N/A	The PRS was correlated with drug-induced QTc prolongation and was a significant predictor of drug-induced Torsade de Pointes in an independent validation sample.
Turkowski et al. (2020) [88]	N/A	423	European	QTc	61	N/A	N/A	The PRS predicted higher QTc values in probands and those with prolonged QTc, but did not predict symptomatic status or serious arrhythmic events.
Nauffal et al. (2022) [89]	84,630	TOPMed— 26,976	European	QTc	1,110,494	Mean QTc change per decile of PRS: 1.4 ms (95% CI 1.3–1.5)	N/A	The PRS showed a significant, independent association with QTc duration: each decile increase in PRS corresponded to a 1.4 ms QTc prolongation, and individuals in the top decile had an average 8.7 ms longer QTc, similar to the effect of monogenic rare variants.
Lancaster et al. (2024) [90]	978	N/A	European	QTc	465,399	OR per one-SD increase: 1.10 (95% CI 1.05–1.16)	AUC 0.74 (95% CI 0.62–0.87)	The PRS predicted both the magnitude of ΔQTc and the likelihood of extreme response, but not post-sotalol QTc > 500 ms.
Simon et al. (2024) [92]	-	2500	Discovery: European Test: Mixed-ancestry	QTc	Not specified	OR per SD: 1.34 (95% CI 1.17–1.53), adjusted model with age, AF, HF, and high-risk medications	AUC for clinical risk tool 0.729 (no 95% CI reported)	The PRS was an independent predictor of diLQTS after exposure to known QT-prolonging drugs. It was an independent predictor after adjusting for age, atrial fibrillation, heart failure, and high-risk medications.
							AUC for clinical risk tool + PRS 0.751 (no 95% CI reported)	
Lahrouchi et al. (2020) [83]	11,546	N/A	European and Japanese	QT	68	OR per SD increase EUR: 1.38 (95% CI 1.30–1.47) JAP: 1.51 (95% CI 1.36–1.67) Meta-analysis:1.41 (95% CI 1.34–1.48)	N/A	The PRS was an independent predictor of LQTS susceptibility in both European and Japanese cohorts and was notably higher in genotype-negative than genotype-positive patients, but it did not independently predict QTc duration in multivariable models or LAE occurrence.
Lopez-Medina et al. (2025) [91]	Used Strauss et al. (2017) study	6970	Mixed-ancestry (self-reported White, African American, and Asian)	QT	61	OR for White patients per SD 1.44 (95% CI 1.09–1.89), adjusted for propensity score OR did not reach statistical significance for African American (OR 2.18, 95% CI 0.98–5.49) and Asian (OR 3.21, 95% CI 0.69–16.87) cohorts	N/A	The PRS significantly predicted marked QTc prolongation with high-risk drug use in self-reported White patients, with suggestive but non-significant associations in African American and Asian groups, likely due to smaller sample sizes.

## 5. Conclusions and Future Directions

**Figure 3:**
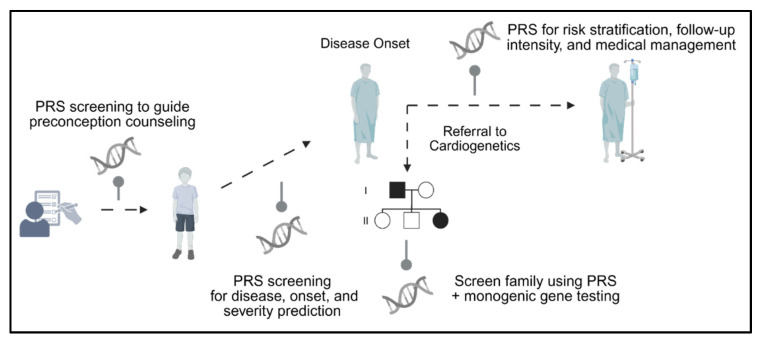
Opportunities for implementation of PRS testing in individuals at risk across their lifespan. PRS can be introduced to guide preconception counselling, to predict risk and severity in early childhood before disease manifestation, at disease onset for risk stratification, and during family genetic screening. PRS: polygenic risk score.

Inherited arrhythmias and cardiomyopathies have traditionally been approached as monogenic disorders. Advances in genomics have revealed a far more nuanced architecture. Rare, high-penetrance variants remain critical for diagnosis and clinical management, but they represent only part of the heritable risk. GWASs have uncovered hundreds to millions of common variants that modify disease susceptibility, penetrance, and severity. PRSs, which integrate these common variants into a quantitative measure of susceptibility, offer the potential to refine risk stratification, particularly in gene-negative individuals or among carriers with variable expressivity. PRS testing in the future could be implemented at various stages in high-risk individuals across their lifespans (Figure 3).

Despite these advances, the clinical implementation of PRS in inherited arrhythmias and cardiomyopathies faces several challenges [92,93,94]. The first is ancestry-specific performance. Most GWASs and resulting PRS are derived from European populations, limiting their predictive accuracy in diverse populations. Second, absolute risk prediction remains imprecise, with PRS generally improving stratification but it does not yet provide definitive guidance for management. On the other hand, biological validation of GWAS loci remains challenging, especially since most hits are in the non-coding genome.

Looking forward, several avenues will be critical for advancing the field. First, functional validation of GWAS loci through cellular models, CRISPR-based editing, and human induced pluripotent stem cell-derived cardiomyocytes (hiPSC-CMs) will bridge statistical associations and mechanistic understanding (Figure 2B) [42,95,96]. There have been large advancements in the ability to dissect the molecular and functional consequences of genetic variants using hiPSC-CMs, with several studies demonstrating feasibility for the assessment of hypercontractility, Ca^2+^ mishandling and arrhythmogenesis, metabolic disruption, and transcriptomic changes in HCM using patient derived hiPSC-CMs carrying the same genetic variants causal for HCM [97]. These advances will be critical for validation of GWAS targets in a human model, along with investigation in animal models for whole-organism validation. Second, inclusion of diverse populations in GWASs is essential to ensure equitable PRS development, given the current predominance of European-ancestry cohorts [98]. Moreover, longitudinal cohort studies will be required to establish the predictive value of PRSs for clinical outcomes and to integrate these scores into decision-making algorithms alongside traditional risk factors. As sample sizes continue to grow, including through large biobank efforts and international consortia, the resolution of GWASs will improve, allowing discovery of smaller-effect loci and better-powered PRS. Multi-ancestry studies will address current limitations in generalizability, while longitudinal cohort studies will link polygenic risk with clinical outcomes over the course of life [99].

Altogether, our understanding of the genetic architecture of inherited arrhythmias and cardiomyopathies has been largely transformed through rapid advances in genomics, particularly GWAS. It is now clear that these disorders are genetically complex and exist on a spectrum influenced by rare and common genetic variation, environmental exposures, and epigenetic regulation. GWASs and PRSs provide a framework for understanding this complexity, refining risk prediction, uncovering novel biology, and ultimately enabling precision cardiovascular medicine.

## Data Availability

No new data were created or analyzed in this study.
